# Resilience to Climate Change by Biocontrol Yeasts Against Ochratoxin A Production in Robusta Coffee

**DOI:** 10.3390/toxins17030110

**Published:** 2025-02-27

**Authors:** Claudia López-Rodríguez, Carol Verheecke-Vaessen, Caroline Strub, Angélique Fontana, Tagro Guehi, Sabine Schorr-Galindo, Angel Medina

**Affiliations:** 1 Magan Centre of Applied Mycology, Cranfield University, Cranfield MK43 0AL, UK; lopez.claudia@deusto.es (C.L.-R.); c.verheecke@cranfield.ac.uk (C.V.-V.); 2 Qualisud, Univ Montpellier, CIRAD, Institut Agro, IRD, Avignon Univ, Univ de La Réunion, 34095 Montpellier, France; caroline.strub@umontpellier.fr (C.S.); angelique.fontana@umontpellier.fr (A.F.); sabine.galindo@umontpellier.fr (S.S.-G.); 3 Faculty of Health Sciences, University of Deusto, 48007 Bilbao, Spain; 4 Laboratory of Microbiology and Molecular Biology, Department of Food Science and Technology, University of Nangui Abrogoua, P.O. Box 801 Abidjan 02, Côte d’Ivoire; g.tagro09@gmail.com

**Keywords:** biocontrol, fungi, mycotoxin, *Coffea canephora*, safety, postharvest control, antifungal yeasts, climate adaptation

## Abstract

*Aspergillus carbonarius* is the main producer of Ochratoxin A (OTA) in coffee. In the last few years, there has been an increasing interest in using yeast isolates as Biocontrol Agents to prevent OTA production in coffee cherries during the primary postharvest processing. Little is known about how climate change abiotic conditions of increased temperature (+2–4 °C), elevated CO_2_ (existing levels of 400 vs. 1000 ppm), and increased drought stress will impact biocontrol resilience. This study examined the effect of a three-way interaction between temperature (27, 30, and 33 °C) x water activity (a_w_) (0.90 and 0.95 a_w_) x CO_2_ level (400 vs. 1000 ppm) on the growth and OTA production of *A. carbonarius* and the resilience of three yeast strains’ biocontrol capacity on fresh coffee cherries. High a_w_ (0.95), CO_2_, and temperature levels increased the production of OTA by *A. carbonarius*. All the yeast biocontrol strains significantly reduced *A. carbonarius* growth by at least 20% and OTA production by up to 85%. From the three strains used, the *Meyerozyma caribbica* strain (Y4) showed the best resilience to climate change, since it reduced both growth (50%) and OTA production (70%) under future scenarios of CO_2_ and a_w_ at all temperatures tested, and should be the one selected for pilot scale experiments in Ivory Coast.

## 1. Introduction

In 2021/2022, 10.1 million tonnes of coffee were produced worldwide [1]. The main food safety concern linked with coffee is contamination with Ochratoxin A (OTA) [2,3]. This mycotoxin, produced by some species of the genera *Aspergillus* and *Penicillium* has both acute and potential chronic toxic effects on humans and animals [4,5]. The health impact of OTA ingestion may include mutagenicity, possible carcinogenicity (group 2B by the International Agency for Research on Cancer (IARC)), hepatoxicity, and nephrotoxicity [6]. To prevent exposure, the maximum regulated levels established by the European Union (EU) range between 3 and 5 μg/kg for roasted coffee beans and soluble coffee, respectively [7]. These limits are especially important in coffee imported from Africa, where concentrations can exceed the maximum regulation up to 10.9 µg/kg of coffee [8].

The main fungal producers of OTA on coffee sourced from Ivory Coast are *A. carbonarius* and *A. ochraceus* [8]. The growth and OTA production of these species can be influenced by environmental conditions such as temperature, water activity (a_w_), and the concentration of atmospheric CO_2_ [9,10]. By the year 2100, global temperatures are predicted to be 2.1–3.5 °C warmer than today, the frequency of droughts is expected to rise, and CO_2_ levels could reach 1000 ppm, according to the Shared Socioeconomic Pathway (SSP) 2–4.5 [11]. The interaction between these factors has been previously studied to evaluate their influence on the growth and production of OTA by *A. carbonarius* on stored coffee beans from Kuwait [12]. The authors reported a 60% increase in OTA production by *A. carbonarius* under elevated CO_2_ (1000 ppm) compared to 400 ppm CO_2_ levels (at 35 °C).

The use of biocontrol microorganisms during coffee postharvest processing has shown the capacity to modulate the final OTA level [13,14]. Microorganisms such as yeast have shown promising results in the reduction of fungal growth and OTA production during coffee fermentation, thanks to their resistance to variable environmental conditions and their lack of production of spores or toxic metabolites [15,16]. Yeasts are part of coffee’s endogenous mycobiota and play a crucial role in breaking down coffee mucilage while regulating the presence of detrimental microorganisms [17,18]. Some of them, such as some of the species belonging to the genera *Saccharomyces*, *Pichia*, or *Hanseniaspora* are considered potential Biocontrol Agents (BCAs) thanks to their ability to reduce the growth and OTA production of ochratoxigenic fungi [19]. Recent results reported the great capacity of three yeasts identified as *Rhodosporidiobolus ruineniae*, *Meyerozyma caribbica*, and *Vishniacozyma* sp. to reduce fungal growth and OTA production by up to 85 and 90% in vitro [20].

The use of BCAs to reduce OTA production under the double effect of temperature and a_w_ has been previously explored in coffee-based media. Neves et al. [15] identified a strain of *S. cerevisiae* CCMA0159 capable of reducing growth and OTA production by 80 and 70%, respectively, for all toxigenic strains. Also, this strain showed good resilience by maintaining its inhibitory capacity under temperatures and a_w_ between 18 and 28 °C and 0.95 and 0.99 a_w_ [15]. However, nothing is known of these BCAs’ resilience under the triple effect of temperature, a_w_, and elevated CO_2_ on fresh coffee cherries. Thus, these findings could have a broader impact on global coffee production sustainability in the future under expected climatic conditions.

This study aimed to identify potential climate change-resilient BCAs against *A. carbonarius* and OTA production in coffee cherries. In the present work, we studied the effect of the three-way interacting climate-related abiotic factors (temperature (27–33 °C) x drought stress (0.90–0.95 a_w_) x existing (400 ppm) and elevated (1000 ppm) CO_2_) in fresh coffee cherries on (i) *A. carbonarius* growth and OTA production and (ii) the BCAs’ resilience to reduce *A. carbonarius* growth and OTA production under climate change scenarios.

## 2. Results

### 2.1. Effect of Climate Change on Aspergillus carbonarius Growth

The growth score was assessed on a scale of 1 to 5, where a rating of 1 denoted minimal growth, and 5 corresponded to the highest level of growth observed (Figure S1). All samples inoculated with *A. carbonarius* only were assigned a growth score of 5, showing a consistent capacity of *A. carbonarius* for growth across all the temperature, a_w_, and CO_2_ levels tested.

### 2.2. Effect of Biocontrol Agent and Abiotic Factors on Fungal Growth

Co-inoculation of *A. carbonarius* with the BCAs reduced fungal growth under some scenarios in comparison to the control (Figure S1). Y1 and Y2 did not impact fungal growth except at lower a_w_ (0.90 a_w_), while the yeast Y4 decreased the growth of *A. carbonarius* at both a_w_ levels. The most important factors reducing growth were the type of strain and the a_w_. Factors such as CO_2_ or temperature did not impact growth. The strains Y1 and Y2 demonstrated comparable outcomes, diminishing the growth to level 3 at 0.90 a_w_, with no reduction in growth observed at 0.95 a_w_. The strain Y4 was the most successful in reducing *A. carbonarius* growth to level 2 at 0.90 a_w_ and to level 4 at 0.95 a_w_.

### 2.3. Effect of Climate Change on A. carbonarius Ochratoxin A Production

**Figure 1:**
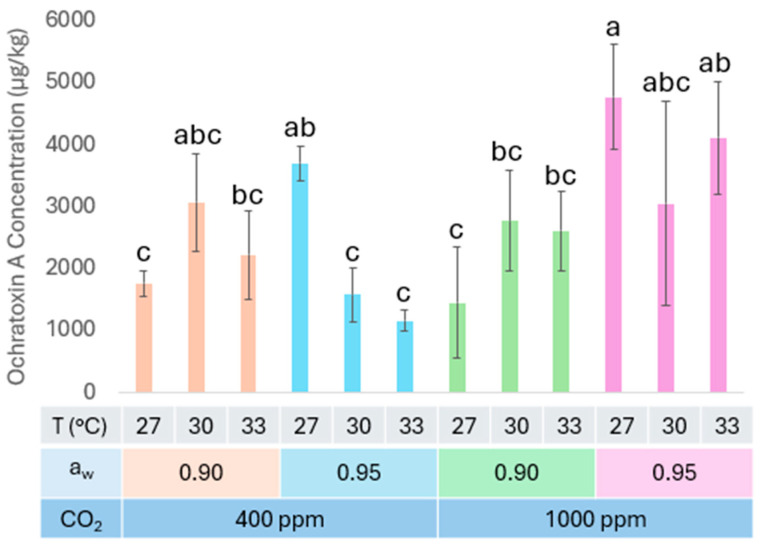
Effect of CO_2_, water activity (a_w_) and temperature (T, °C) on the Ochratoxin A concentration on fresh coffee inoculated with *A. carbonarius*. Different letters indicate significant differences (*p* > 0.05) between different temperatures at a specific a_w_ and CO_2_ using the Fit Least Squares Method and LSMeans differences Tukey HSD. Bars represent the average and the error bars standard deviation.

Under all scenarios, *A. carbonarius* produced a concentration of at least 1000 µg of OTA per kg of coffee. The a_w_ (*p* < 0.05) and CO_2_ (*p* < 0.05) factors significantly impacted the production of OTA by *A. carbonarius* (Table S1; Figure 1). An increase in a_w_ significantly increased OTA production only at 27 °C, as observed in Figure 1. Current CO_2_ conditions (400 ppm CO_2_) showed lower OTA production compared to elevated CO_2_ (1000 ppm) at 33 °C and 0.95 a_w_. The comparison of the current (400 ppm CO_2_, 0.95 a_w_, and 27 °C) and future (1000 ppm CO_2_, 0.90 a_w_, and 33 °C) combined climatic factors showed consistent OTA production by *A. carbonarius* in both conditions.

### 2.4. Efficiency of BCAs on Ochratoxin A in Climate Change Conditions

The impact of BCAs on OTA concentration was further investigated jointly with the three-way interacting climate change factors. The factors of strain, a_w_, and temperature (T) and the interacting factors T* CO_2_, CO_2_* a_w_ *T, BCA*T, BCA* CO_2_*a_w_, and BCA* CO_2_*T* a_w_ have a significant effect (*p* < 0.05) on the percentage of OTA reduction (Table S2).

In Table 1, the significance of the effect of abiotic factors on each yeast strain capacity for OTA reduction using the Fit Least Squares model was analyzed. For all strains, a_w_ (*p* < 0.05) had a significant effect on their capacity to control OTA concentration, while for strain Y4, temperature and CO_2_ also had an effect on OTA reduction (*p* < 0.05).

**Figure 2:**
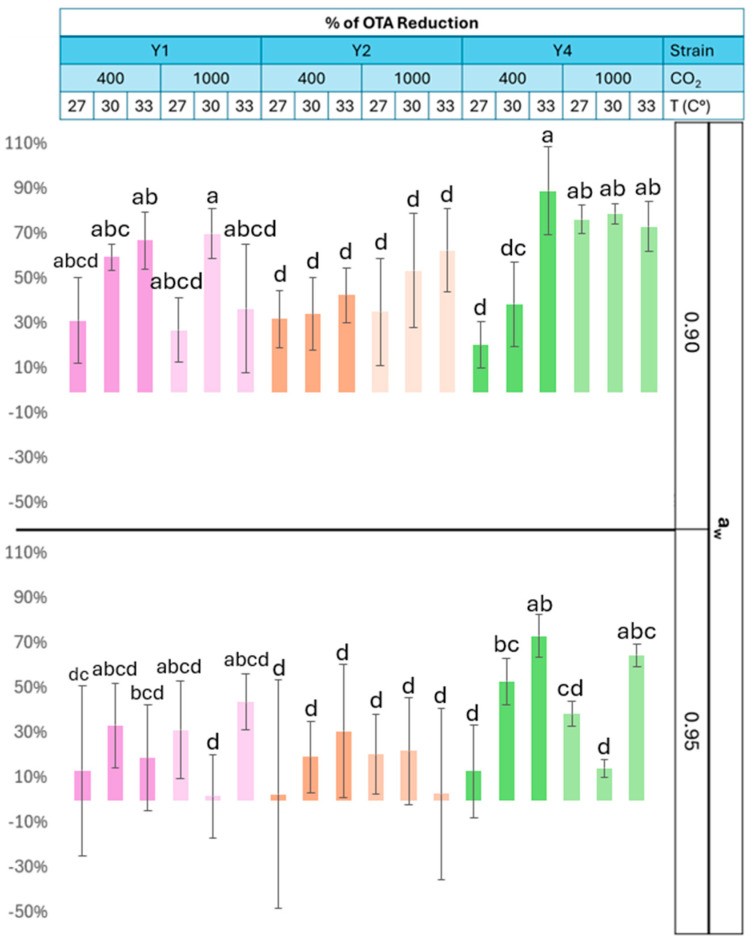
Effect of CO_2_, temperature, and water activity on the % reduction in OTA concentration in fresh coffee cherries. Different letters indicate significant differences (*p* > 0.05) in the % reduction in OTA of each strain, using the Fit Least Squares method and Tukey HSD test. Bars represent AV ± SD.

As shown in Figure 2, under current CO_2_ conditions and 0.90 a_w_, there is a significant reduction in the OTA concentration (*p* < 0.05) at 33 °C by the strains Y1 and Y4 (Figure 2). At 0.95 a_w_, Y4 reduces significantly (*p* < 0.05) OTA at 30 and 33 °C. At 33 °C and both a_w_ levels, Y4 reduced OTA production by at least 70%, while the Y1 and Y2 strains reduced OTA by a maximum of 70 and 40%, respectively. Under future CO_2_ conditions and 0.90 a_w_, there is a decrease of OTA concentration of 60 and 80% at 33 °C for Y2 and Y4, respectively. At 0.95 a_w_, the reduction by the Y1 and Y4 strains is 30% at 27 °C and 40% and 60% at 33 °C for each strain, respectively. At these same conditions, Y2 shows a weaker ability to reduce OTA, of 20% and 5%, respectively, for 27 and 33 °C.

Higher CO_2_ and temperature promote the reduction of OTA by the strains Y2 and Y4 at 0.90 a_w_. At the same a_w_ but current CO_2_ levels, OTA reduction is higher at 33 °C for all the strains. From all studied combinations of CO_2_, temperature, and a_w_, all strains reduced OTA production by at least 10%, except Y1 at 1000 ppm CO_2_, 30 °C, and 0.95 a_w_ and Y2 at 400 ppm CO_2_, 27 °C, and 1000 ppm CO_2_, and 33 °C at 0.95 a_w_.

## 3. Discussion

This study addressed for the first time how the triple effect of temperature, a_w_, and CO_2_ levels impact the efficiency of different yeasts as potential BCAs against growth and the production of OTA by *A. carbonarius* on fresh coffee beans. Previous studies only analyzed the effect of a_w_ and temperature (not considering the effect of CO_2_) in vitro on coffee-based media, using mostly *Aspergillus* section *Circumdati* strains and without the inoculation of potential BCAs [21]. The first results from Estrada-Bahena et al. analyzed OTA contamination of coffee under a range of a_w_ levels between 0.108 and 0.821 during storage, concluding that at 0.82 a_w_, the contamination of OTA in coffee cherries increased [22]. Oliveira et al. [23] confirmed these results with an OTA risk index of the species *A. carbonarius* between 2.5 and 3.5 times higher at 0.95–0.99 a_w_ and 25–32 °C than at 0.92 a_w_ and 20 °C. The authors used a model based on 11 assays performed under a variety of 0.91 to 0.99 a_w_ levels and temperatures of 20–38 °C.

In the present work, a_w_ was the most important abiotic factor increasing *A. carbonarius* fungal growth. Previous studies have described the optimal growth of *A. carbonarius* between 0.93 and 0.96 a_w_ in a coffee-based medium [15], which aligns with our results. Surprisingly, temperature did not influence fungal growth, while in other studies it has been positively correlated with higher *A. carbonarius* growth [24].

The strain Y4 was the most efficient BCA at reducing *A. carbonarius* growth, which is in alignment with Lopez-Rodriguez et al. [20]. Many studies have shown the effect of yeasts on *A. carbonarius* fungal growth [17,24,25]. Neves et al. [15] isolated *S. cerevisiae* CCMA 0159 from coffee beans and analyzed its growth inhibitory effect on *A. carbonarius* in coffee-based media. The results showed that 28 °C and 0.99 a_w_ were the optimum conditions for fungal growth inhibition by *S. cerevisiae* CCMA 0159, while in our experiment, a higher a_w_ did not give any advantage to the capacity of the yeasts to reduce fungal growth.

Regarding OTA production, Akbar et al. previously studied OTA production of *A. carbonarius* under different conditions of CO_2_, temperature, and a_w_ on a coffee-based medium [12]. That study concluded that elevated CO_2_ (1000 ppm) and temperatures (35 °C) increased OTA production by 40%. In our study, under elevated CO_2_ and a_w_, there was an increase in OTA production of 70% at 33 °C compared to the same conditions at ambient CO_2_. This suggests that under future temperatures that are expected to rise towards 30 °C in coffee-producing areas [26], the OTA concentration may increase two-fold in coffee. Concerning a_w_, higher a_w_ showed a correlation with slightly higher OTA production by *A. carbonarius*, which was significant at 27 °C. Previous studies have also shown that an a_w_ level above 0.95 a_w_ positively influenced OTA production levels by *A. carbonarius* [23].

The present study strain Y4 presented the best resilience, since it reduced OTA production under more scenarios. Previous studies have shown that yeast species such as *S. cerevisiae* can reduce the production of OTA during the confrontation against *A. carbonarius* on different food matrixes and under different climatic conditions. For example, Neves et al. [15] researched the strain *S. cerevisiae* CCMA 0159 against *A. carbonarius* on a coffee-based medium and showed a significant reduction of 80% in OTA production at high temperatures (28 °C) and a_w_ (0.99). During this investigation, expression of the OTA biosynthetic genes *pks*, *nrps*, and *p450* belonging to the OTA genic cluster was reduced, potentially correlating with decreased OTA production. Similar results were reported by Cubaiu et al. [24], where *S. cerevisiae* strains isolated from wine were tested against *A. carbonarius*, demonstrating the ability to inhibit OTA production by at least 65% and reducing the expression of the *pks* gene as well. Complementary studies of the proposed BCAs’ effect on the downregulation of OTA biosynthetic gene expression in *A. carbonarius* would be needed. To our knowledge, there has been only one experimental study of a BCA against an ochratoxigenic fungi using coffee cherries as a substrate, which was conducted by De Melo Pereira et al. [27]. In this work, strains of *Pichia fermentans* and *A. westerdijkiae* were inoculated on coffee beans and incubated for 10 days at 28 °C and an a_w_ of 0.63 at the beginning and 0.97 a_w_ at the end of the incubation period. The results showed a reduction in OTA production by 88% in interaction with *P. fermentans*. These results align with Y4’s capacity to reduce OTA up to 80% depending on the climate scenarios. This confirms the uniqueness of our BCA efficiency trials of *A. carbonarius* on fresh coffee cherries, highlighting both the novel selection of biocontrol agent species—previously untested for this purpose—and the selection of the substrate.

It should be noted that the two studies previously mentioned did not monitor the experiments under controlled temperature, a_w_, or CO_2_ conditions. Also, there are very few publications using BCAs, such as bacteria or filamentous fungi, in coffee-based media or directly on coffee cherries [21,27]. Most of the studies applying BCAs on coffee focus on the use of *Pseudomonas* sp. and *Trichoderma* sp. for the treatment of coffee plant infections, such as coffee rust or coffee wilt disease [28,29,30,31].

Finally, we recommend the study of the proposed BCAs in confrontation with other strains of *A. carbonarius* and other strongly ochratoxigenic species, such as *A. westerdijkiae*. This would help to find out the spectrum of OTA reduction of these BCAs to tackle OTA contamination through their application in field trials.

## 4. Conclusions

This multifactorial study has shown that the use of BCAs such as *Rhodosporidiobolus ruineniae* (Y1), *Vishniacozyma* sp. (Y2), and *Meyerozyma caribbica* (Y4) in co-culture with a high OTA-producing strain of *A. carbonarius* on coffee cherries, could result in a reduction of up to 50 and 90% of fungal growth and OTA production, respectively. Thus, these BCAs represent an alternative to creating a more sustainable and safe coffee postharvest chain.

The efficiency of the BCAs was affected by temperature, a_w_, and CO_2_, resulting in a higher efficiency of OTA reduction under future conditions of elevated CO_2_ and temperature and reduced a_w_. *M. caribbica* (Y4) is proposed as the most efficient BCA, and the application of this BCA on fresh coffee cherries could reduce contamination with OTA by at least 70% under future conditions of CO_2_ and a_w_, representing an adaptable BCA whose optimized formulation could lead to its commercialization.

## 5. Materials and Methods

### 5.1. Coffee Sampling

Nineteen kilograms of fresh Robusta coffee cherries, which were not processed, were harvested by hand in 2020 using sterilized gloves, placed in Stomacher bags, and stored and transported at 4 °C. Upon reception, all samples were then kept at −20 °C. The coffee was cultivated in Akoupé, Côte d’Ivoire (6° 23′ north, 3° 54′ west) at an altitude of 126 m. The coffee was irradiated to eliminate the microbiota while conserving the biochemical composition, as previously described for other foodstuffs [32]. This process started by placing the coffee in a Petri dish without a cover inside a Biosafety cabinet equipped with UV-C (254.7 nm wavelength) lamps. The coffee was irradiated with UV-C for 1 h and 15 min and turned every 15 min to eliminate homogenously the native microbiota on its surface. The efficacy of the disinfection step was previously tested in fresh coffee without UV-C treatment (0 h UV-C), with 0.5 h of UV-C treatment (0.5 h UV-C), 1 h of UV-C treatment (1 h UV-C), and 1 h and 15 min (1.25 h UV-C), totaling four treatments. Afterwards, the coffee was directly plated on PDA and MRS media for fungal and bacterial growth, respectively, and incubated for 72 h. The disinfection with UV-C for 1 h and 15 min proved to be the most effective with 100% reduction of fungal and bacterial growth when compared to the non-irradiated coffee.

### 5.2. Yeasts and Fungal Strains

Three yeasts previously isolated from Ivorian Robusta coffee cherries were used [20]. Based on phenotypic characteristics, biochemical tests, and sequence analyses of the ITS1 and ITS2 regions and the D1/D2 domains of the 26S rRNA gene, the three yeasts were identified as *Rhodosporidiobolus ruineniae* (Y1), *Vishniacozyma* sp. (Y2), and *Meyerozyma caribbica* (Y4), as described by Lopez-Rodriguez et al. [20]. These strains were selected based on their ability to reduce OTA concentration by up to 90%. The native ochratoxigenic strain of *A. carbonarius* (D3) used in this study was isolated from Ivorian Robusta coffee cherries and identified based on phenotypic characteristics and sequence analyses of the ITS1 and ITS2 regions and the partial regions of the β-tubulin and Calmodulin genes. It was selected for its high OTA production capacity and used as a toxigenic pathogen in the present study [20].

### 5.3. Inoculation of Coffee Beans

The yeast strains were sub-cultured on PDB (Potato Dextrose Broth) and incubated in agitation for 48 h at 25 °C. Then, the cells were counted using a counting chamber (Neubauer improved, Paul Marienfield, GE) and suspended in a 1% (w-v) Tween^®^80 solution. From this solution, a specific volume was added to reach a final adjusted concentration of 10^6^ cells/g of fresh coffee cherries. The ochratoxigenic strain was sub-cultured on PDA (Potato Dextrose Agar). After 7 days at 25 °C, the spores were removed from the colony using a sterile loop and transferred to a 1% (w-v) Tween^®^80 solution. Similarly, from this solution, a specific volume was added to reach a final concentration of 10^3^ spores/g of fresh coffee. The final stock solution was used to modify the a_w_ of the cherries.

### 5.4. Incubation Conditions and Elevated CO_2_ Treatment

Fifteen grams of fresh coffee cherries were aseptically distributed into sterile glass culture vessels of a 100 mL capacity (Magenta™, Sigma, St. Louis, MO, USA) with a vented lid (10 mm with polypropylene membrane 0.22 µm pore size) to allow gas exchange but keep the environment inside the vessel sterile. Then, 2 or 12 mL of sterile water was added to reach 0.90 or 0.95 a_w_, respectively, based on the moisture adsorption curve of coffee. After adding the water, the jars were manually shaken twice to ensure good rehydration and were kept at 4 °C for 24 h for full adsorption and equilibration. The next day, the jars were inoculated with 1 mL of inoculum, either yeast and fungus, or only fungus, depending on the treatment, and equally shaken to ensure the inoculum soaked all the cherries. All treatments were run in five replicates. The jars were placed in 12 L airtight containers and incubated in a dynamic climatic chamber (SANYO Electric Co., Ltd., MLR-350H, Tokyo, Japan) at three different temperatures (27, 30, and 33 °C) and the Climate Change (CC) scenario of the Southern East region of Ivory Coast. The temperature was monitored by introducing a thermometer inside the boxes. The containers included a beaker (300 mL) of water with glycerol (vegetable origin, >99%) at 0.90 and 0.95 a_w_ to minimize moisture loss. They were flushed twice a day for 12 min either with current (400 ppm) or increased (1000 ppm) CO_2_ concentrations by using calibrated gas cylinders (British Oxygen Company, Guildford, Surrey, UK) with a flow rate of 3 L/min to renew 3-fold the air volume of the incubation containers [33]. During the flush, the outlet hose was opened to ensure flow of the air. After 12 min, the outlet and inlet hoses were closed. After 7 days, the samples were collected and oven dried at 65 °C for 48 h. The samples were visually assessed for fungal growth, and a scoring was assigned according to Figure S1 before storage at 4 °C. The scoring system ranges from 1 to 5, with higher scores indicating increased fungal growth (Figure S1).

### 5.5. Ochratoxin A Quantification

The samples were processed according to Malachova et al. [34]. Briefly, the samples were ground using a laboratory blender with a stainless-steel blade (Waring, Stamford, CT, USA). Five grams of sample were extracted with 20 mL of extraction solvent (acetonitrile/water/acetic acid 79:20:1, *v*/*v*/*v*). The samples were extracted for 90 min using a VWR VX-2500 Multitube Vortexer (VWR, Radnor, PA, USA) at 1800 rpm speed and subsequently centrifuged for 5 min at 3000 rpm (Thermo Scientific Sorvall ST40 Centrifuge, Loughborough, UK). The extracts were transferred into glass vials and diluted with the same volume of dilution solvent (acetonitrile/water/acetic acid 20:79:1, *v*/*v*/*v*).

Quantification was performed using a qTRAP-LC-MS/MS 6500+ system (Exion Series) without further pre-treatment. An ACE 3-C18 column (2.1 × 100 mm, 3 µm particle size; Hichrom, Berkshire, UK) equipped with a C18 security guard cartridge (4 × 3 mm, Gemini Agilent, Oxford, UK) kept at 60 °C was used for chromatographic separation with the following gradients: Solvent A: water:methanol:acetic acid (89:10:1, *v*:*v*:*v*) and Solvent B: methanol:water:acetic acid (97:2:1, *v*:*v*:*v*), both supplemented with 5 mM ammonium acetate, which was applied for 20 min. The flow rate of the mobile phase was 0.6 mL/min, and the injection volume was set to 1 µL. MS/MS was performed in scheduled multiple reaction monitoring (MRM) with a 60 s window. Analyst version 1.6.3 (AB SCIEX™, Framingham, MA, USA) and MultiQuant version 3.0.3 (AB SCIEX™, Framingham, MA, USA) were used for data acquisition and analysis, respectively, following the method described in Malachova et al. [34].

### 5.6. Data Analysis

Statistical analysis was performed using the statistical package JMP^®^ Pro 18 (SAS Institute Inc., Cary NC, USA). Shapiro–Wilk tests were used to determine normality and Levene’s test to assess the equality of variance. The OTA production data did not follow normal distribution, and logarithmic and square root transformations did not sufficiently normalize the data for analysis. Consequently, the General Linear Model (GLM), specifically the Standard Least Squares model was selected, and the parametric Fit Least Square Method (*p* = 95%) was applied to analyze the effect of the factors CO_2_, a_w_, and T. The factors followed an LS Means Tukey HSD test to assess potential differences across treatments. The variable used in the analysis was either OTA production to assess the impact of abiotic factors on *A. carbonarius*’s OTA production or the percentage reduction in OTA to evaluate the effect of the biocontrol strains on *A. carbonarius*’s OTA production. Both variables were analyzed following the Standard Least Squares model.

## Figures and Tables

**Table 1:** Summary of the statistical analysis for the effect of interacting climate-related abiotic factors on % of reduction of Ochratoxin A by each BCA after 7 days. The probability values in bold were significant (*p* < 0.05). Results based on Fit Least Squares Method for the effects of water activity (a_w_), temperature (T), CO_2_ level, and BCA.

Factors	DF	*p*-Value (Prob > F)	Factors	DF	*p*-Value (Prob > F)
		OTA (µg/kg)			OTA (µg/kg)
BCA: Y1					
a_w_	1	**<0.0001**	T* CO_2_	2	0.4169
T	2	0.0625	T*a_w_	2	**0.0179**
CO_2_	1	0.6508	CO_2_*a_w_*T	2	**0.0039**
a_w_ *CO_2_	1	0.3699			
BCA: Y2					
a_w_	1	**0.0007**	T* CO_2_	2	0.6580
T	2	0.3874	T*a_w_	2	0.7202
CO_2_	1	0.4482	CO_2_*a_w_*T	2	0.2504
a_w_ *CO_2_	1	0.2782			
BCA: Y4					
a_w_	1	**<0.0001**	T* CO_2_	2	**<0.0001**
T	2	**<0.0001**	T*a_w_	2	0.2624
CO_2_	1	**0.0074**	CO_2_*a_w_*T	2	**<0.0001**
a_w_ *CO_2_	1	**<0.0001**			

## Data Availability

The data used to support the findings of this study can be made available by the corresponding author upon request.

## Supplementary Materials

The following supporting information can be downloaded at: https://www.mdpi.com/article/10.3390/toxins17030110/s1. Figure S1: Examples of the five different levels of growth used to assess the growth level of *A. carbonarius* on the coffee cherries and the inoculum and a_w_ conditions during the experiment; Table S1: Summary of the statistical analysis for the effect of interacting climate-related abiotic factors on Ochratoxin A production by *A. carbonarius* after 7 days; Table S2: Summary of the statistical analysis for the effect of interacting climate-related abiotic factors and BCA on Ochratoxin A % of reduction by *A. carbonarius* after 7 days.

## Abbreviations

The following abbreviations are used in this manuscript:

BCA	Biocontrol Agent
OTA	Ochratoxin A
IARC	International Agency for Research on Cancer
CO_2_	Carbon dioxide
a_w_	Water activity
