# Supplementary material for: Resilience to Climate Change by Biocontrol Yeasts Against Ochratoxin A Production in Robusta Coffee
Source: Toxins (Basel). 2025 Feb 27;17(3):110. doi: 10.3390/toxins17030110 (PMC11945309; doi:10.3390/toxins17030110)
Supplement: Supplementary file 1 [file toxins-17-00110-s001.zip › toxins-3465055-supplementary.pdf]

Supplementary Materials: Resilience to Climate Change by Biocontrol Yeasts Against Ochratoxin A Production in Robusta Coffee

Claudia López-Rodríguez, Carol Verheecke-Vaessen, Caroline Strub, Angélique Fontana, Tagro Guehi, Sabine Schorr-Galindo and Angel Medina

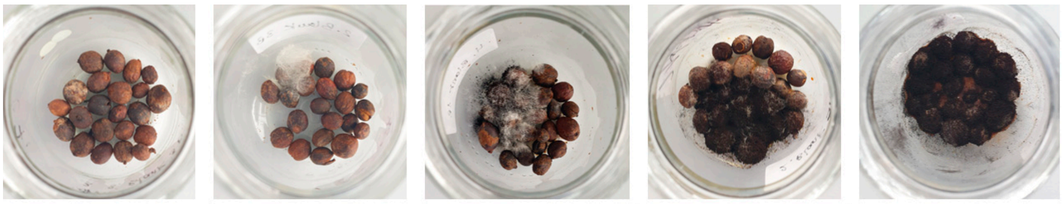

1	2	3	4	5
Blank (0.90 a_w)	Blank (0.95 a_w)	--	--	--
	Y4 (0.90 a_w)	Y1 (0.90 a_w)<br>Y2 (0.90 a_w)	Y4 (0.95 a_w)	Y1 (0.95 a_w)<br>Y2 (0.95 a_w)<br>Control

Figure S1. Examples of the five different levels of growth used to assess the growth level of *A. carbonarius* on the coffee cherries and the inoculum and a_w conditions during the experiment. CO_2 and temperature levels were not included because they did not have any effect on the growth level. Increasing growth from level 1 to 5. Dimensions of the glass vessel are 77 x 77 x 97 mm.

Table S1. Summary of the statistical analysis for the effect of interacting climate-related abiotic factors on ochratoxin A production by *A. carbonarius* after 7 days. The probability values in bold were significant (p<0.05). Results based on Fit Least Squares Method for the effects of water activity (a_w), temperature (T) and CO_2 level.

Factors	DF	*p*-value (Prob > F) OTA (µg/kg)	Factors	DF	*p*-value (Prob > F) OTA (µg/kg)
**a_w**	**1**	**0.0020***	T* CO_2	2	0.0576
T	2	0.3458	T*a_w	2	**<0.0001***
CO_2	1	**0.0004***	CO_2*a_w*T	2	0.5691
a_w*CO_2	1	**0.0001***			

Table S2. Summary of the statistical analysis for the effect of interacting climate-related abiotic factors and BCA on ochratoxin A % of reduction by *A. carbonarius* after 7 days. The probability values in bold were significant (p<0.05). Results based on Fit Least Squares Method for the effects of water activity (a_w), temperature (T), CO_2 level, and BCA.

Factors	DF	*p*-value (Prob > F) OTA (µg/kg)	Factors	DF	*p*-value (Prob > F) OTA (µg/kg)
**BCA**	**2**	**<0.0001***	BCA* CO_2	2	0.3175
a_w	1	**<0.0001***	BCA *T	4	**0.0260***
T	2	**<0.0001***	BCA *a_w	2	0.6656
CO_2	1	0.21241	BCA * CO_2*a_w	2	**0.0290***
a_w*CO_2	1	0.0540	BCA *T*a_w	4	0.1193
T* CO_2	2	**0.0067***	BCA*CO_2*T*a_w	4	**0.0094***
T*a_w	2	0.1856	CO_2*a_w*T	2	**0.0028***

The *p*-value (Prob > F) was calculated from the values of F ratio and DF (degrees of freedom) of each effect.
